# Hospital falls clinical practice guidelines: a global analysis and systematic review

**DOI:** 10.1093/ageing/afae149

**Published:** 2024-07-18

**Authors:** Jonathan P McKercher, Casey L Peiris, Anne-Marie Hill, Stephen Peterson, Claire Thwaites, Sally Fowler-Davis, Meg E Morris

**Affiliations:** La Trobe University Academic and Research Collaborative in Health (ARCH), and CERI, Bundoora, VIC, 3086, Australia; La Trobe University Academic and Research Collaborative in Health (ARCH), and CERI, Bundoora, VIC, 3086, Australia; Allied Health, The Royal Melbourne Hospital, Parkville, VIC, 3050, Australia; School of Allied Health, The University of Western Australia, Perth, WA, 6000, Australia; La Trobe University Academic and Research Collaborative in Health (ARCH), and CERI, Bundoora, VIC, 3086, Australia; La Trobe University Academic and Research Collaborative in Health (ARCH), and CERI, Bundoora, VIC, 3086, Australia; The Victorian Rehabilitation Centre, Healthscope, Glen Waverley, VIC, 3150, Australia; School of Allied Health and Social Care, Anglia Ruskin University, Chelmsford, United Kingdom; La Trobe University Academic and Research Collaborative in Health (ARCH), and CERI, Bundoora, VIC, 3086, Australia; The Victorian Rehabilitation Centre, Healthscope, Glen Waverley, VIC, 3150, Australia

**Keywords:** accidental falls, falls prevention, clinical practice guidelines, hospital, systematic review, older people

## Abstract

**Background:**

Hospital falls continue to be a persistent global issue with serious harmful consequences for patients and health services. Many clinical practice guidelines now exist for hospital falls, and there is a need to appraise recommendations.

**Method:**

A systematic review and critical appraisal of the global literature was conducted, compliant with the Preferred Reporting Items for Systematic Reviews and Meta-Analyses guidelines. Web of Science, Embase, CINAHL, MEDLINE, Epistemonikos, Infobase of Clinical Practice Guidelines, Cochrane CENTRAL and PEDro databases were searched from 1 January 1993 to 1 February 2024. The quality of guidelines was assessed by two independent reviewers using Appraisal of Guidelines for Research and Evaluation Global Rating Scale and Appraisal of Guidelines of Research and Evaluation Recommendation Excellence (AGREE-REX). Certainty of findings was rated using Grading of Recommendations Assessment, Development and Evaluation Confidence in Evidence from Reviews of Qualitative Research. Data were analysed using thematic synthesis.

**Results:**

2404 records were screened, 77 assessed for eligibility, and 20 hospital falls guidelines were included. Ten had high AGREE-REX quality scores. Key analytic themes were as follows: (i) there was mixed support for falls risk screening at hospital admission, but scored screening tools were no longer recommended; (ii) comprehensive falls assessment was recommended for older or frail patients; (iii) single and multifactorial falls interventions were consistently recommended; (iv) a large gap existed in patient engagement in guideline development and implementation; (v) barriers to implementation included ambiguities in how staff and patient falls education should be conducted, how delirium and dementia are managed to prevent falls, and documentation of hospital falls.

**Conclusion:**

Evidence-based hospital falls guidelines are now available, yet systematic implementation across the hospital sector is more limited. There is a need to ensure an integrated and consistent approach to evidence-based falls prevention for a diverse range of hospital patients.

## Key Points

- Comprehensive falls assessment during hospital admission was advocated for older patients, those with frailty or multimorbidity, after a fall or when there was marked change in medical status.
- The use of scored falls risk screening tools was not recommended in high-quality recent guidelines.
- Individual interventions such as patient education, staff training, use of assistive devices, exercise, environmental adaptations and safe footwear were consistently recommended to prevent hospital falls.
- Multifactorial falls prevention interventions were agreed to be of benefit and varied according to individual needs.
- Barriers to implementing guidelines included absence of specific guidance for delivering patient and staff falls education and uncertainties about how to manage delirium and dementia to reduce falls. There was limited protocol-driven activity and a failure to adapt falls guidelines to the needs of people from culturally diverse backgrounds.

## Background

Hospital falls and associated injuries are a significant worldwide problem [1, 2]. Hospital falls can result in life-threatening injuries, head trauma, fractures and even death [1, 3, 4]. They can also have significant psychological effects such as loss of confidence, depression or anxiety [5, 6]. Hospital falls–related injuries are predicted to increase across the globe due to escalating population ageing [2, 7]. Older people have a higher prevalence of falls risk factors such as frailty, multimorbidity, visual impairment and impaired balance [2]. Falls rates in acute hospitals typically range from 3 to 7 falls per 1000 bed days [8–10], increasing to 4–14 falls per 1000 bed days in rehabilitation hospitals [3, 11, 12]. Systematic implementation of evidence-based guidelines is needed to help reduce the frequency and impact of hospital falls, especially in older people who are the major recipients of hospital care [2].

Clinical practice guidelines are derived from research evidence and aim to facilitate the rapid implementation of evidence into clinical practice to improve patient outcomes [13]. Hospital falls guidelines aim to guide health services to systematically mitigate, manage and document falls and associated injuries by providing best-practice recommendations for health services, health professionals and patients [13, 14]. They can also be used to inform policies at local, state, national and international levels, and to guide health professional clinical decision-making [13]. Montero-Odasso *et al*. [5] reviewed clinical practice guidelines on falls prevention for older adults in home, community, hospital and aged care settings. At the time of that review, they found strong agreement across guidelines regarding falls prevention in the home and community, yet limited consensus on mitigating hospital falls [5]. Also, their review did not examine falls prevention guidelines for people <65 years of age. Williams-Roberts *et al*. [15] reviewed hospital guidelines on falls risk screening and assessment yet did not analyse recommendations for mitigating, managing or documenting hospital falls.

Several existing hospital clinical practice guidelines have been recently updated and new ones have been published. Given these new developments, the aim of this global review was to identify, appraise, analyse, compare and synthesise published clinical practice guidelines on hospital falls prevention, management and documentation.

## Methods

The review was conducted in accordance with Preferred Reporting Items for Systematic Reviews and Meta-Analyses (PRISMA) recommendations [16]. It was registered with the international prospective register of systematic reviews (PROSPERO) and followed the published protocol [17, 18].

### Eligibility criteria

Clinical practice guidelines were included if they were published guidelines on hospital falls prevention, management or documentation. To be included, the guideline needed to be for adult patients and have at least one recommendation specific to the prevention or management of falls in a hospital setting. Condition-specific clinical practice guidelines were included if they met these criteria.

Guidelines were excluded if they were restricted solely to falls prevention in patients under the age of 18 years, given that management of the paediatric population is different and there are stand-alone paediatric guidelines [19]. Guidelines were also excluded if they did not contain the word ‘hospital’ or if they pertained to occupational falls, sport-related falls or falls that occurred outside a hospital and presented to a hospital emergency department for management of a community-based fall. Publications were also excluded from the analysis if they were policies or systematic reviews, theses, not available in full text or published >30 years ago, which was before publication of a pioneering guideline by Simpson *et al*. [20].

Falls policies, clinical standards and toolkits for hospital falls prevention and management were excluded and will be reviewed in separate manuscripts. A policy is a broad statement of goals that affords a general framework for activity [21] (such as the EuroSafe policy) [22]. Standards are concise quality statements that outline the care patients should receive for a specific clinical condition [23, 24] (e.g. the Australian NSQHS standards [25] or the UK NICE quality standards) [26]. Hospital toolkits are resource repositories for existing policies, standards, clinical guidelines and educational materials [27] and are exemplified by the Agency for Healthcare Research and Preventing Falls in Hospitals toolkit [28] and the CDC Stopping Elderly Accidents, Deaths, and Injuries (STEADI) falls toolkit [29].

### Search strategy and selection criteria

Search terms were related to international clinical practice guidelines, hospital and falls prevention. A senior research librarian (MI) was consulted and reviewed the search strategy. Nine databases were searched: Web of Science, Embase, CINAHL, MEDLINE, Epistemonikos, Infobase of Clinical Practice Guidelines, Cochrane CENTRAL and PEDro from 1 January 1993 until 1 February 2024, with search strategies modified accordingly (Supplementary Table 1). Grey literature was searched, including via the following websites: National Institute for Health and Care Excellence (NICE), Australian Commission on Safety and Quality in Health Care (ACSQHC), and National Health and Medical Research Council Clinical Practice Guidelines. Reference lists of included papers and previous systematic reviews of falls prevention guidelines were hand searched to identify publications not found in the primary search [5, 15]. We also contacted researchers specialising in hospital falls to identify any guidelines not retrieved during the prior searches.

Identified articles were imported into Covidence and duplicates were removed [30]. Titles, abstracts and retained full-text records were independently screened by two authors (J.P.M., C.T.), and the inclusion criteria were applied to determine eligibility. Reasons for exclusion were recorded. Disagreements were resolved through group discussion with a third reviewer (M.E.M.).

A data extraction sheet was purposefully designed to collect data related to guideline details (first author, year, country), hospital falls prevention recommendations, patient engagement in design or implementation of guidelines, falls, data collection and falls documentation methods. For any guideline that had targeted more than one setting, data were only extracted on recommendations pertaining to the hospital setting. Two independent researchers (J.P.M., C.T.) extracted and entered the data.

### Falls guideline quality appraisal

Quality for each of the included guidelines was independently assessed by two authors (J.P.M., C.T.) using the Appraisal of Guidelines of Research and Evaluation Recommendation Excellence (AGREE-REX) and the Appraisal of Guidelines for Research and Evaluation Global Rating Scale (AGREE GRS) [31, 32]. The two reviewers met to reach agreement on rating scores. Remaining differences were resolved by consultation with the third reviewer (M.E.M.). The AGREE-REX assessed the credibility of recommendations, the degree to which consumers were involved in guideline development and the extent to which the guidelines could be implemented [31]. The AGREE GRS tool included four key assessment areas: style of presentation, development process, completeness of guideline reporting and clinical validity [32].

### Critical analysis and thematic synthesis

A thematic synthesis was completed by two reviewers in NVivo (J.P.M., C.T.) and checked by a third expert (M.E.M.) [33]. The synthesis was conducted in three stages as recommended by Cochrane [34] and Thomas and Harden [35]. Using NVivo, line-by-line coding was first completed for each guideline. Then, similar codes were identified from across guidelines and grouped into related topics. The topics were used to formulate descriptive themes that encapsulated the meaning of a set of codes. The descriptive themes were then interpreted to create new analytical themes that went beyond the coded content [35]. Throughout this process, each emergent theme was scrutinised, refined and agreed upon by our review team (J.P.M., C.T., M.E.M.).

### Confidence in cumulative evidence

Three members of the research team (J.P.M., C.T., M.E.M.) with experience in qualitative systematic reviews assessed the confidence of the review findings using the Grading of Recommendations Assessment, Development and Evaluation Confidence in Evidence from Reviews of Qualitative Research (GRADE-CERQual) (Table 3) [36]. The GRADE-CERQual was used to analyse the relevance, adequacy of data, coherence and methodological limitations of the review’s findings. Confidence in the evidence for these elements was rated from very low to high [36].

## Results

### Included studies

**Figure 1:**
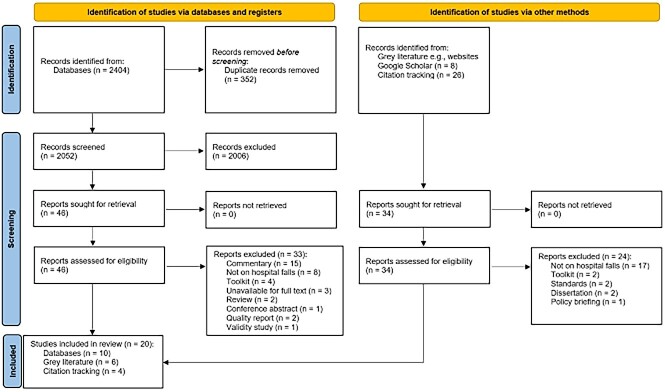
PRISMA flow diagram [16].

A total of 2404 studies were found during the database searches. Following automated duplicate removal [18], 2052 studies were screened for title and abstract. The full text of 77 studies were assessed for eligibility, of which 57 were excluded. In total, 20 guidelines were included in the review (Figure 1). The rationale for exclusion of works at full-text review is summarised in Supplementary Table 2.

### Quality appraisal

The AGREE GRS domain mean scores were high across all 20 guidelines ranging from 6.15 to 6.65 on a 7-point scale (Table 1). Domain 4 (clinical validity) scored the highest across the guidelines. The other three AGREE GRS domains (process of development, presentation style and completeness of reporting) also had high mean scores. The AGREE-REX overall score was high (mean 82.09%) yet varied considerably across the included guidelines. Ten of the guidelines had an overall score >95%. Six scored <60%, four of which were published prior to 2013. These guidelines had limited incorporation of the values and preferences of target users, consumers, patients or policy-makers [31].

**Table 1:** Quality appraisal using the AGREE-REX and AGREE GRS [31, 32]

Guideline (year, country)	AGREE-REX overall score, %	AGREE GRS domain scores, 7-point scale				AGREE GRS overall assessment, 7-point scale		
		1: Process of development	2: Presentation style	3: Completeness of reporting	4: Clinical validity	Quality of the guideline	I would recommend this guideline	I would use this guideline in my professional decisions
SF [37] (2024, Australia)	100	7	7	7	7	7	7	7
Kim *et al*. [38] (2023, South Korea)	100	7	7	7	7	7	7	7
SIGN [39] (2023, UK)	100	7	7	7	7	7	7	7
Montero-Odasso *et al*. [7] (2022, Global)	100	7	7	7	7	7	7	7
Schoberer *et al*. [40] (2022, Austria)	100	7	7	7	7	7	7	7
WHO [41] (2021, Global)	100	7	7	7	7	7	7	7
Cho *et al*. [42] (2020, South Korea)	55.6	7	5	7	5	5	5	5
Rimland *et al*. [43] (2017, EU)	96.3	7	6	7	7	7	7	7
RNAO [44] (2017, Canada)	98.1	7	7	7	7	7	7	7
Kruschke [45] (2017, USA)	37.0	1	6	1	6	5	6	6
Gea *et al*. [46] (2015, Spain)	88.9	5	7	6	6	6	6	6
Belita *et al*. [47] (2013, England)	85	7	7	7	7	7	7	7
NICE [48] (2013, UK)	100	7	7	7	7	7	7	7
AGILE [49] (2012, UK)	51.9	5	7	5	7	6	7	7
Degelau *et al*. [50] (2012, USA)	74.1	5	5	4	6	6	6	6
WI RHA [51] (2011, USA)	98.1	7	7	7	7	7	7	7
ACSQHC [52] (2009, Australia)	96.3	7	7	7	7	7	7	7
SG MOH [53] (2006, Singapore)	51.2	5	6	6	6	6	6	6
AGS [54] (2001, USA)	59.3	6	6	7	7	6	6	6
Simpson *et al*. [55] (1998, UK)	50.0	5	5	5	6	5	5	5
Mean score	82.09	6.15	6.5	6.25	6.65	6.45	6.55	6.55

*Abbreviations*: SF, Stroke Foundation; SIGN, Scottish Intercollegiate Guidelines Network; WHO, World Health Organisation; RNAO, Registered Nurses’ Association of Ontario; NICE, National Institute for Health Care Excellence; AGILE, Chartered Physiotherapists Working with Older People; WI RHA, Winnipeg Regional Health Authority; ACSQHC, Australian Commission on Safety and Quality in Health Care; SG MOH, Singapore Ministry of Health; AGS, American Geriatrics Society.

### Characteristics of included guidelines

Eight of the guidelines applied to adults of any age [37–39, 41, 42, 44, 46, 50]. Twelve were solely for older people, usually defined as ≥65 years [7, 40, 41, 43, 45, 48, 49, 51–55]. All of the falls guidelines that we reviewed originated from high-income countries. Four originated from the USA [45, 50, 51, 54], four from UK [39, 48, 49, 55], two from South Korea [7, 41] and two were global guidelines [7, 41]. We also reviewed guidelines from the European Union [43], Spain [46], Australia [37, 52], Canada [44] and Singapore [53] (Table 1). Eighteen guidelines were published in English [7, 37–41, 43–45, 47–55], one in Korean [42] and one in Spanish [46].

### Analytic themes

Five main analytic themes were identified: (i) half of the guidelines still recommended falls risk screening on hospital admission, but calculating falls risk scores on hospital admission was no longer recommended; (ii) comprehensive falls assessment was frequently recommended for hospitalised older patients, patients who fell during the admission or those with complex needs, with some uncertainty about when to undertake this assessment; (iii) single and multifactorial falls interventions were consistently recommended; (iv) patient engagement in the design or implementation of guidelines was inconsistent; (v) barriers to implementation related to ambiguities about how delirium and dementia are best managed to prevent hospital falls; lack of details about falls documentation; and limited recommendations for best practice around how best to deliver staff and patient education. Supplementary Tables 3–8 provide representative quotations from the guidelines supporting these themes.

#### (i) Screening

Falls risk screening typically occurs on Day 1 of hospital admission to quickly identify patients at high risk (Supplementary Table 3) [15, 56]. In contrast, a comprehensive falls assessment is a more detailed process that can take up to an hour and is used to identify all of the risk factors for an individual with more complex needs [7]. Falls risk screening at admission was not universal. It was recommended in 10/20 included guidelines [7, 40, 44, 46, 48–53, 55] with most advocating for this to occur within the first 24 h after admission [7, 40, 44, 46, 48–53, 55]. Only older guidelines published before 2011 recommended the use of scored falls risk screening/stratification assessment tools, known as FRATs [51–53]. FRATs assign risk scores pertaining to the estimated likelihood of the patient falling and clinicians then select mitigation strategies based on the score. Recent guidelines [7, 40] recommended that scored FRATs should not be routinely used in hospitals [7, 40, 46, 48, 53]. This is because all hospitalised older adults are at a high risk of falling [48], and risk factors differ from one hospital setting to another due to differences in case mix (Supplementary Table 3) [46, 52, 53]. FRAT scoring can be problematic as health professionals can underestimate the falls risk and then assign mitigation strategies that do not match patient needs [57, 58]. Along these lines, the Johns Hopkins hospital FRAT was recently shown to have poor discrimination between fallers and non-fallers and underestimated falls risk [59]. To quote Montero-Odasso *et al*. [7], ‘There is a case for divesting from fall risk screening tool scoring in the hospital setting as it does not reduce falls and takes valuable time.’

#### (ii) Comprehensive falls assessment

Comprehensive falls assessment for older patients was mentioned in 17/20 guidelines [7, 40, 42, 44–46, 48–55]. However, guidelines differed about when to do this assessment. Some advised that only patients deemed to be a high falls risk during initial screening should be assessed further [44, 49, 51, 52, 54]. Others recommended comprehensive assessment of all older people and any adult patient after a hospital fall or marked change in medical status (Supplementary Table 4) [7, 44, 53].

#### (iii) Single and multifactorial interventions

As seen in Table 2, patient education, staff education and environmental modifications were the single interventions most supported by clinical guidelines. These three interventions were recommended in 12/20 guidelines [7, 40–42, 44, 46, 48, 50–54]. For example, Gea *et al*. [46] advised: ‘Provide relevant oral and written information and support to the patient and their families and carers... (explain) the patient’s personal risks of falling during their stay in the hospital; teach the patient how to use the call bell and encourage them to use it if they need help; give consistent messages about when a patient should ask for help before getting up or move; help the patient participate in any intervention designed to address his or her personal risks; ensure that relevant information is shared between different services.’ As seen in Supplementary Table 5, recommendations also consistently included medication review, exercise (especially strength, mobilisation, balance training), osteoporosis management, nutritional therapy (especially vitamin D) and continence management [7, 40–46, 48–55]. Recent guidelines did not recommend the use of bed and chair alarms, non-slip socks and physical restraints [7, 41, 45]. Some of these interventions were mentioned in older clinical guidelines published before 2011 [51–54].

**Table 2:** Recommendations included in clinical practice guidelines

Areas		Clinical practice guidelines																			
		SF [37]	Kim [38]	SIGN [39]	Montero-Odasso [7]	Schoberer [40]	WHO [41]	Cho [42]	Rimland [43]	RNAO [44]	Kruschke [45]	Gea [46]	Belita [47]	NICE [48]	AGILE [49]	Degelau [50]	WI RHA [51]	ACSQHC [52]	SG MOH [53]	AGS [54]	Simpson [55]
**Falls risk screening**						**✓**				**✓**	**✓**	**✓**	**✓**	**✓**	**✓**	**✓**	**✓**	**✓**	**✓**		**✓**
Falls history^a^										**✓**	**✓**	**✓**	**✓**	**✓**	**✓**	**✓**					
Gait and balance^a^										**✓**		**✓**	**✓**	**✓**		**✓**	**✓**	**✓**			
Scored screening tools					**X**	**X**						**X**		**X**			**✓**	**✓**	**✓**		
**Comprehensive falls assessment**				**✓**	**✓**	**✓**		**✓**		**✓**	**✓**	**✓**		**✓**	**✓**	**✓**	**✓**	**✓**	**✓**	**✓**	**✓**
Gait and balance		**✓**		**✓**	**✓**	**✓**		**✓**		**✓**	**✓**	**✓**	**✓**	**✓**	**✓**	**✓**	**✓**	**✓**	**✓**	**✓**	**✓**
Cardiovascular					**✓**								**✓**	**✓**			**✓**	**✓**			
Delirium, dementia or cognitive impairment					**✓**							**✓**	**✓**	**✓**			**✓**	**✓**			
Footwear				**✓**	**✓**		**✓**					**✓**		**✓**			**✓**	**✓**			**✓**
Vision and hearing		**✓**		**✓**	**✓**							**✓**	**✓**	**✓**			**✓**	**✓**		**✓**	
**Multifactorial interventions**		**✓**			**✓**	**✓**	**✓**	**✓**	**✓**	**✓**	**✓**	**✓**		**✓**	**✓**	**✓**	**✓**	**✓**	**✓**	**✓**	**✓**
Education					**✓**	**✓**	**✓**	**✓**		**✓**		**✓**		**✓**		**✓**	**✓**	**✓**	**✓**		
Environment adaptations					**✓**	**✓**		**✓**		**✓**		**✓**		**✓**		**✓**	**✓**	**✓**	**✓**	**✓**	**✓**
Medication review				**✓**	**✓**	**✓**				**✓**		**✓**	**✓**	**✓**		**✓**			**✓**	**✓**	
Exercise		**✓**	**✓**	**✓**	**✓**	**✓**									**✓**		**✓**		**✓**	**✓**	**✓**
Osteoporosis					**✓**												**✓**	**✓**			
Vitamin D				**✓**	**✓**					**✓**		**✓**			**✓**		**✓**	**✓**		**✓**	
Continence management					**✓**									**✓**			**✓**	**✓**	**✓**		
**Guideline implementation**					**✓**	**✓**				**✓**						**✓**			**✓**		**✓**
Co-design			**✓**		**✓**																

**✓** = recommended, **X** = not recommended. *Abbreviations*: SF, Stroke Foundation; SIGN, Scottish Intercollegiate Guidelines Network; WHO, World Health Organisation; RNAO, Registered Nurses’ Association of Ontario; NICE, National Institute for Health Care Excellence; AGILE, Chartered Physiotherapists Working with Older People; WI RHA, Winnipeg Regional Health Authority; ACSQHC, Australian Commission on Safety and Quality in Health Care; SG MOH, Singapore Ministry of Health.

a Falls history can be screened at admission and a more detailed falls history is also often ascertained during a comprehensive falls assessment, especially for older patients or those with frailty or multimorbidity.

Multifactorial interventions were supported in 17/20 guidelines (Table 2) [7, 40–46, 48–55]. Multifactorial interventions were advised to be individualised for each patient based on findings of screening or comprehensive assessment (Supplementary Table 6) [7, 41, 44, 53, 60]. According to the 2022 world falls guidelines for older adults: ‘… We recommend that personalised single or multidomain falls prevention strategies based on identified risk factors or behaviours or situations should be implemented for all hospitalised older adults (≥65 years of age), or younger individuals identified by the health professionals as at risk of falls (grade 1C acute care and 1B sub-acute)’ [7].

#### (iv) Patient engagement in guideline design and implementation

The thematic analysis showed that patient engagement in the design or implementation of guidelines was inconsistently reported. Only two publications specified the inclusion of hospital patients or their families in the co-design of falls guidelines [7, 38]. These sought the perspectives and preferences of older adults in the development of their guidelines [7, 38]. Many guidelines mentioned that stakeholder engagement was a key determinant of success even though they did not describe exactly how this could be achieved [37, 48, 51, 52, 55]. For example, ACSQHC [44] stated: ‘Older people themselves are at the centre of the guidelines. Their participation, to the full extent of their desire and ability, encourages shared responsibility in health care, promotes quality care, and focuses on accountability’ [52]. Yet, procedures to achieve this were not elucidated.

#### (v) Barriers to guideline implementation

The thematic analysis identified some barriers to guideline implementation (Supplementary Table 8). Delirium management was mentioned as a key challenge, and ‘When delirium, dementia and cognitive impairment are managed well, falls are less prevalent. Adapting the environment to promote safety and educating caregivers in strategies for safe mobility can also be of benefit in older adults with delirium’ [7]. Yet, only five of the guidelines included specific procedures for managing delirium, dementia or cognitive impairment to prevent falls [7, 50–53].

Our thematic analysis also showed that guideline implementation was limited by inconsistencies or a lack of clarity about exact methods for educating patients and staff about hospital falls (Supplementary Table 5), even though 12/20 guidelines mentioned this was important [7, 40–42, 44, 46, 48, 50–54]. The clinical practice guideline by Schoberer *et al*. [40] stated: ‘Patients at risk of falling should be informed about fall risks and receive training and advice regarding fall prevention measures.’ Yet, the methods to implement this were not given. The Singapore Ministry of Health gave more details and recommended: ‘Education programmes should be targeted at health-care providers, patients and care-givers… Education programmes for patients and family/caregivers should include: risk factors for falls; safe mobilisation and limitations to activities; safety precautions in the ward and ward orientation; importance of staying active and being mobile unless contraindicated.’ [53] Modes of education were not specified in detail.

Another barrier to implementation pertained to documentation of hospital falls, which varied widely (see Supplementary Table 7). Whereas the WHO (2021) recommended ‘Monitor administrative and clinical data (e.g. falls per 1000 occupied bed days, fall rates by type of falls, specific location of falls) and investigate the frequency and severity of falls (e.g. injury rate, injury rate by severity)’ [41], it was not clear what platforms should be used to collect falls data and how staff could access the data. Because disparate systems are used and data are often manually entered, many falls incidents are not adequately addressed and safety data are sometimes wasted [61].

## Discussion

This paper analysed 20 hospital clinical practice guidelines spanning 30 years of evidence changes and found high-quality evidence for several interventions to prevent and manage hospital falls (Table 2). Changes in falls guidelines over this period reflect the rapid growth of clinical research on falls risks and mitigation [1, 3, 11, 14, 57, 62, 63]. For example, older hospital guidelines recommended the use of scored FRATs in an attempt to predict who is most at risk of falling [64, 65]. In contrast, more recent high-quality guidelines recommend against using FRATs as they were found to be unreliable at predicting who will fall in hospital [7, 40, 46, 48]. Hospital populations have also changed over the last 30 years, so the earlier logic of screening to identify the exceptionally less mobile patient makes less sense in more recent times where the majority of patients are highly dependent. Older guidelines did not mention patient engagement in guideline development or implementation [38–55], whereas recent ones did [7, 38]. Older falls guidelines sometimes recommended the use of chair alarms [53], restraints [51] or grip socks [50], whereas more recent ones do not [7, 41], due to limited evidence or evidence of harm [66, 67]. Professional and societal attitudes to physical restraints and chair alarms have also changed over the last 30 years [68], leading to divestment from some of these interventions. As the negative physical effects of these restrictive interventions on patients’ conditioning and function are now better understood, there is a need to move towards multidisciplinary strategies that promote patient independence and physical activity [69].

**Table 3:** GRADE-CERQual summary of qualitative findings [36]

Summary of key review findings	Studies contributing to the review finding	Confidence assessment	Explanation of CERQual assessment
Routine use of scored falls risk screening tools (FRATs) was not recommended to prevent hospital falls	[51–53]	High	Minor methodological limitations, relevance, adequacy and coherence concerns
Comprehensive falls assessment was recommended to prevent hospital falls in older patients or those with frailty, multimorbidity, hospital falls or changes in medical status	[7, 40, 42, 44–46, 48–55]	High	Minor methodological limitations, relevance, adequacy and coherence concerns
Single interventions including patient and staff education, environment adaptation, exercise (mobility, strength and balance training), osteoporosis management, nutritional therapy, vitamin D supplementation, continence management, management of delirium and dementia, footwear and medication review were recommended	[7, 40–46, 48–55]	High	Minor methodological limitations, relevance, adequacy and coherence concerns
Multifactorial interventions were recommended to prevent hospital falls	[7, 40–46, 48–55]	High	Minor methodological limitations, relevance, adequacy and coherence concerns
Organisational support facilitated successful implementation of falls prevention and management recommendations	[7, 40, 44, 50, 53, 55]	High	Minor methodological limitations, relevance, adequacy and coherence concerns
Systematic documentation of falls-related data was recommended yet there was a lack a clarity on platforms and procedures to do this	[41, 42, 46, 53]	Moderate	Moderate adequacy concerns. Minor methodological limitations, relevance and coherence concerns

For older hospital patients, recent clinical guidelines consistently recommended that health professionals conduct comprehensive falls assessments [7, 40, 42, 44–46, 48–55] although it is unclear when this should occur during the hospital admission. It was also unclear how the workforce could assess every older person, given that most hospital patients are over the age of 60 years and staffing is finite [56]. Recent high-quality guidelines also support the implementation of single interventions or multifactorial interventions to prevent and manage falls. This is in agreement with the meta-analysis by Morris *et al*. [1], a Cochrane review by Cameron *et al*. [3] and the WHO falls statement [41]. Single interventions supported by guidelines included patient and staff falls prevention education, use of assistive devices, fast responses to call bells, safe footwear, environmental modifications, exercise (e.g. strength and balance training), medication management and evidence-based management of delirium and dementia [7, 37–42, 44, 46, 48–55]. Multifactorial approaches incorporating two or more of these interventions [7, 40–46, 48–55] were corroborated by a Cochrane review by Cameron *et al*. [3].

Patient education was identified as one of the most effective methods of reducing falls and injuries, particularly when hospital procedures were implemented to foster close collaboration between patients, families and health professional teams [56, 70, 71]. When staff implement patient education very soon after hospital admission, patient engagement in falls prevention is enhanced [11, 56, 63]. In hospitals, this can sometimes be difficult due to competing demands on staff time. To address this challenge, workforce redesign has been recommended, such as using supervised assistants to assist with falls education [56]. A recent trial by Morris *et al*. [56] showed that using trained allied health assistants to deliver scripted falls education in the first 48 hours after hospital admission was feasible and of benefit.

Unlike prior reviews [5, 15], we evaluated the extent to which patients were involved in clinical guideline development. Patient-centred care is now a key pillar of healthcare [72, 73], and it was surprising that only two of the included falls guidelines mentioned patients, families or members of the public in the design, evaluation or updating of guidelines. This is arguably a serious gap because most falls prevention strategies in hospitals directly rely on patient engagement (e.g. patient falls education, patient call-bell activation for assistance with toileting, patients engaging in exercise, wearing safe footwear and using prescribed mobility devices). Our review raises the need to address power imbalances between patients and health providers to improve patient experiences and care [72]. Developing falls guidelines and policies in consultation with consumer groups makes it more likely that guidelines will meet the needs and expectations of patients and other target users [74–76]. There is also a need to prioritise ongoing updates to hospital falls prevention policies and to support hospital management and key stakeholders to implement them [77].

Effective implementation of evidence-based hospital falls guidelines into clinical practice was recognised as a critical gap in healthcare and occurred at individual and systemic levels [78, 79]. Systemic health service barriers noted in our synthesis related to ambiguities in how to deliver patient falls education, inconsistencies in staff falls prevention training, and a poor understanding of how best to manage dementia, delirium and cognitive impairment, even though these appear to be particularly powerful ways to mitigate hospital falls [7, 40–46, 48–55]. There are now robust dementia and delirium guidelines developed for hospitals that provide patient-centred recommendations, and this conjoint information can guide the implementation of falls prevention activities [80–82]. A recent systematic review and meta-analysis by He *et al*. [83] found that evidence-based delirium management strategies have the potential to reduce the risk of falls in older patients.

A notable barrier to implementation was that few guidelines specified how staff should document, report and access data on hospital falls locally or across hospital networks [84]. Many countries now have key quality indicators, e.g. how hip fractures incurred from hospital falls are reported and analysed [82, 85]. Some countries use data to report against quality metrics, with financial penalties if targets for falls rates and injuries are missed [86]. According to the WHO [41], the implementation of accessible hospital falls data platforms is a high priority for healthcare agencies. As pointed out by Jehu and Skelton [87], further standardisation of measurement is still required [87].

The strengths of this review included a comprehensive search of nine databases, a detailed quality appraisal process using the AGREE GRS and AGREE-REX and thematic synthesis of the literature. We also used the GRADE-CERQual to determine the certainty of our recommendations [36]. Whereas the recent systematic review by Montero-Odasso *et al*. [5] and recent world guidelines [7] were restricted to people 60–65 years and older, our evaluation encompassed all adults in hospital. For the guidelines that we reviewed that targeted all hospitalised adults, it was not possible to partition out recommendations for each age subgroup. Another limitation was that 18/20 guidelines were published in English and all originated from high-income countries. This limits the applicability of the findings to non-English-speaking countries and low- and middle-income countries, which account for 75% of fatal falls according to the WHO [41]. Another limitation was that recommendations in guidelines could have been influenced by the opinions, clinical experience and cultural background of the guideline development group and written as generic indicators of best practice [88]. They might not always apply to individual patients, particularly those from culturally and linguistically diverse backgrounds [88].

## Conclusion

High-quality and consistent hospital falls guidelines are now available worldwide. Next steps should focus on educating and equipping policy-makers, hospital leaders, health professionals, patients and families to ensure rapid uptake into policies, standards, toolkits and daily care. Patients need to be engaged in this process as key participants. There remains a need for more high-quality, original research on hospital falls prevention, as well as translational research that evaluates how to address implementation effectively at the individual, ward and hospital levels.

## Supplementary Material

aa-24-0421-File002_afae149
