# Supplementary material for: Hospital falls clinical practice guidelines: a global analysis and systematic review
Source: Age Ageing. 2024 Jul 18;53(7):afae149. doi: 10.1093/ageing/afae149 (PMC11255989; doi:10.1093/ageing/afae149)
Supplement: aa-24-0421-File002_afae149 [file aa-24-0421-file002_afae149.pdf]

Supplementary Table 1.
Example search strategy

Ovid MEDLINE(R) ALL <1946 to Feb 1, 2024>

#	Searches	Results
1	Hospitals/	103858
2	"inpatient hospital*".mp.	4541
3	"acute hospital*".mp.	7175
4	hospitali?ation.mp.	289540
5	Hospitalization/	138390
6	"acute care".mp.	29225
7	Subacute Care/	1447
8	Inpatients/	30538
9	1 or 2 or 3 or 4 or 5 or 6 or 7 or 8	436653
10	Accidental Falls/	28333
11	fall*.mp.	293138
12	"fall* prevention".mp.	5144
13	"fall* management".mp.	182
14	"fall* management".mp.	182
15	(fall* adj15 injur*).mp. [mp=title, book title, abstract, original title, name of substance word, subject heading word, floating sub-heading word, keyword heading word, organism supplementary concept word, protocol supplementary concept word, rare disease supplementary concept word, unique identifier, synonyms, population supplementary concept word, anatomy supplementary concept word]	16806
16	(fall* adj15 fracture*).mp. [mp=title, book title, abstract, original title, name of substance word, subject heading word, floating sub-heading word, keyword heading word, organism supplementary concept word, protocol supplementary concept word, rare disease supplementary concept word, unique identifier, synonyms, population supplementary concept word, anatomy supplementary concept word]	10101
17	10 or 11 or 12 or 13 or 14 or 15 or 16	293138
18	Practice Guideline/	31087
19	Practice Guidelines as Topic/	127837
20	"clinical practice guideline*".mp.	21419
21	"clinical guideline*".mp.	19224
22	Guideline Adherence/	35194
23	Evidence-Based Medicine/	76302

24	"best practice guideline*".mp.	2486
25	"consensus statement".mp.	7242
26	18 or 19 or 20 or 21 or 22 or 23 or 24 or 25	270673
27	9 and 17 and 26	214
28	limit 27 to dt=20230801-20240201	2

Supplementary Table 2.
Excluded at full text review

Author or organisation	Year	Title	Reason for exclusion
Lung Foundation Australia [1]	2024	The COPD-X Concise	Not on hospital falls
Agency for Healthcare Research and Quality [2]	2024	Preventing falls in hospital toolkit	Toolkit
EuroSafe [3]	2024	Injuries among older people	Policy briefing
Australian Commission on Safety and Quality in Healthcare [4]	2023	The National Safety and Quality Health Service Standards	Clinical governance standards
Camicioli et al. [5]	2023	Prevention of Falls in Parkinson's Disease: Guidelines and Gaps	Not on hospital falls
Callus et al. [6]	2022	The development of an in-patient fall acute management guideline	Conference abstract
Ellen et al. [7]	2022	Global guidelines for falls in older adults: Working group 12: Fear of falling	Review
Todd et al. [8]	2022	Clinical Practice Guideline for Preventing Falls in Geriatric Patients	Dissertation
Carroll Smith [9]	2022	Fall Clinical Practical Guideline in a Psychiatric Hospital	Dissertation
American Diabetes Association [10]	2022	Professional Practice Committee: Standards of Medical Care in Diabetes 2022	Not on hospital falls
Ebpracticenet (BE): Working Group Development of Primary Care Guidelines [11]	2021	Fall prevention in elderly persons with an increased risk of falling (Occupational therapy guideline)	Not on hospital falls
Fraenkel et al. [12]	2021	2021 American College of Rheumatology Guideline for the Treatment of Rheumatoid Arthritis	Not on hospital falls

Cho et al. [13]	2020	Development of ICNP-based inpatient falls prevention catalogue	Toolkit
College of Occupational Therapists [14]	2020	Occupational therapy in the prevention and management of falls. Practice guideline	Not on hospital falls
Johnson et al. [15]	2020	Patient fall risk and prevention strategies among acute care hospitals	Reliability and validity study
Lamb et al. [16]	2020	Screening and Intervention to Prevent Falls and Fractures in Older People	Not on hospital falls
Ocker et al. [17]	2020	Preventing Falls Among Behavioural Health Patients	Quality improvement report
Araki et al. [18]	2020	Japanese clinical practice guideline for diabetes 2019	Not on hospital falls
Kolasinski et al. [19]	2019	2019 American College of Rheumatology/Arthritis Foundation Guideline for the Management of Osteoarthritis of the Hand, Hip, and Knee	Not on hospital falls
Kim et al. [20]	2019	Clinical Practice Guideline for Cardiac Rehabilitation in Korea: Recommendations for Cardiac Rehabilitation and Secondary Prevention after Acute Coronary Syndrome	Not on hospital falls
The Danish Health Authority [21]	2018	National clinical guideline for the prevention of falls in elderly people	Not on hospital falls
Malcolm et al. [22]	2018	In-hospital management of diabetes	Not on hospital falls
Kenny et al. [23]	2017	Falls in older adults	Commentary
Kim et al. [24]	2017	Evidence-based guidelines for fall prevention in Korea	Not on hospital falls
Lee et al. [25]	2017	The CDC's STEADI initiative: Promoting older adult health and independence through fall prevention	Commentary

Morris and O'Riordan [26]	2017	Prevention of falls in hospital	Commentary
Pearce [27]	2017	Preventing falls in hospital	Editorial
American Geriatrics Society and British Geriatrics Society [28]	2016	AGS/BGS Clinical Practice Guideline: Prevention of falls in older persons. Summary of recommendations	Not on hospital falls
Williams [29]	2016	Guide to ensure older people avoid trips and falls in hospital	Commentary
Crandall et al. [30]	2016	Prevention of fall-related injuries in the elderly: an Eastern Association for the Surgery of Trauma practice management guideline	Not on hospital falls
Lee et al. [31]	2015	Prevention of fall in the hospital	Commentary
NICE [32]	2015	Falls in older people: assessing risk and prevention	Clinical standard
Barker [33]	2014	Assessment and prevention of falls in older people	Commentary
Keus et al. [34]	2014	European Physiotherapy Guideline for Parkinson's disease	Not on hospital falls
Swift and Iliffe [35]	2014	Assessment and prevention of falls in older people - Concise guidance	Commentary
Tung and Newman [36]	2014	Fall prevention in hospitalized patients	Commentary
van der Marck et al. [37]	2014	Consensus-based clinical practice recommendations for the examination and management of falls in patients with Parkinson's disease	Not on hospital falls
Stevens [38]	2013	The STEADI Tool Kit: A Fall Prevention Resource for Health Care Providers	Toolkit
Stevens and Phelan [39]	2013	Development of STEADI: a fall prevention resource for health care providers	Toolkit

Chow et al. [40]	2012	Optimal preoperative assessment of the geriatric surgical patient: A best practices guideline from the American college of surgeons national surgical quality improvement program and the American geriatrics society	Commentary
Health Care Association of New Jersey [41]	2012	Fall management guideline	Toolkit
Ummenhofer and Suhm [42]	2012	Fractured neck of femur: Guidelines and beyond	Editorial
Vance [43]	2012	The clinical practice guideline for falls and fall risk	Not on hospital falls
American Geriatrics Society and British Geriatrics Society [44]	2011	Summary of the Updated American Geriatrics Society/British Geriatrics Society Clinical Practice Guideline for Prevention of Falls in Older Persons	Not on hospital falls
Beauchet et al. [45]	2011	How to manage recurrent falls in clinical practice: guidelines of the French Society of Geriatrics and Gerontology	Not on hospital falls
Bradley [46]	2011	Falls in older adults	Commentary
Kline et al. [47]	2011	Fall risk assessment and prevention	Quality improvement report
American Geriatrics Society and British Geriatrics Society [48]	2010	AGS/BGS clinical practice guideline: prevention of falls in older persons.	Not on hospital falls
Handoll [49]	2010	Prevention of falls and fall related injuries in older people in nursing homes and hospitals	Commentary
Oliver [50]	2010	Preventing falls and fall-related injuries in Hospitals	Commentary
Naqvi et al. [51]	2009	An evidence-based review of the NICHE guideline for preventing falls in older adults in an acute care setting	Review
Oliver [50]	2007	Preventing falls and falls-injuries in hospitals and long-term care facilities	Commentary

Loew and Maupetit [52]	2005	Preventing falls and fractures today	Commentary
American Medical Directors Association [53]	2003	Falls and fall risk clinical practice guideline	Not on hospital falls
Moreland et al. [54]	2003	Evidence-based guidelines for the secondary prevention of falls in older adults	Not on hospital falls
Carson and Cook [55]	2000	A strategic approach to falls prevention	Toolkit
Feder et al. [56]	2000	Guidelines for the prevention of falls in people over 65. The Guidelines' Development Group	Not on hospital falls
Rutledge et al. [57]	1998	Fall risk assessment and prevention in healthcare facilities.	Commentary
Baraff et al. [58]	1997	Practice guideline for the ED management of falls in community-dwelling elderly persons	Not on hospital falls

Clinical standards: short statements on integral areas of clinical practice for a specific condition where there are variations from best practice;[59] Policy: broad statement of goals that affords an overall framework for activity;[60] Toolkit: resource repositories for existing policies, standards, clinical guidelines and educational materials supporting evidence-based practice.[61]

Supplementary Table 3.
Themes and example quotations for admission screening vs immediate comprehensive assessment

Theme	Example Quotations			
**Falls risk screening on admission (mixed results)**	<u>Supporting rapid screening all patients early after admission:</u><br><br>"Patients… should be **screened for fall risks** when they are admitted to the hospital… but the use of an assessment tool is not recommended."[62] (Schoberer 2022)	<u>Supporting rapid screening all patients early after admission:</u><br><br>"Screen all adults to identify those at risk for falls. **Conduct screening** as part of admission processes, after any significant change in health status… screening should include… identifying a history of previous falls, identifying gait, balance and/or mobility difficulties and using clinical judgement."[63] (RNAO 2017)	<u>Supporting rapid screening all patients early after admission:</u><br><br>"Older people in contact with health professionals **should be asked routinely** whether they have fallen in the past year and asked about the frequency, context and characteristics of the fall/s."[64, 65] (NICE 2013)<br><br>"Do not use fall risk prediction tools to predict inpatients' risk of falling in hospital."[64, 65] (NICE 2013)	<u>Supporting rapid screening all patients early after admission:</u><br><br>"All older adults who are admitted to hospital **should be screened** for their falls risk, and this screening should be done as soon as practical after they are admitted… when a change in health or functional status is evidence, or when the older adult's environment changes."[66] (Winnipeg 2011)
**Falls risk screening on admission (mixed results)**	<u>Supporting early assessment of all older patients, patients with co-morbidities or patients with marked change in health status:</u><br><br>"Regard the following groups of inpatients as being at risk of falling in hospital and manage their care according to recommendations 1.2.2.1 to 1.2.3.2: all patients aged 65 years or older; patients aged 50 to 64 years who are judged by a clinician to be at higher risk of falling because of an underlying condition."[64, 65] (NICE 2013)			

Supplementary Table 4.
Themes and example quotations for comprehensive, multi-factorial falls assessment during the hospital stay

Theme	Example quotations				
**Comprehensive falls assessment**	"A **multifactorial falls risk assessment** for those at high risk of falling, which enables advice for falls prevention and management interventions, includes the following domains: gait and balance, muscle strength, medications, cardiovascular disorders including orthostatic hypotension, dizziness, functional ability and walking aids, vision and hearing, musculoskeletal disorders, foot problems and footwear, neurocognitive disorders… neurological disorders."[67] (World Guidelines for Older People; Montero-Odasso 2022)	"A **multifactorial fall risk assessment** should be offered to older adults who require assistance as a result of a fall, or report several falls in the last year, or present balance problems. The evaluation must be carried out by a trained and experienced professional."[68] (Gea 2015)	"Older people who present for medical attention because of a fall, or report recurrent falls in the past year, or demonstrate abnormalities of gait and/or balance should be offered a **multifactorial falls risk assessment**."[64, 65] (NICE 2013)	"As part of a multifactorial program for adults with increased falls risk in hospital, conduct a **systematic and comprehensive interprofessional falls risk assessment**…"[66] (Winnipeg 2011)	"A **falls risk assessment** should include the following elements: History of falls; medications associated with increased fall risk; secondary or specific diagnoses known to affect falls risk …; Postural hypotension; Seizures; dizziness, vertigo; Altered mental status …; Altered elimination status …; Impaired/ deterioration of activities of daily living ; Impaired mobility or gait; Poor visual acuity; Poor safety awareness; Lack of insight into own health condition; Risk taking behaviour."[69] (Singapore Ministry of Health 2006)

Supplementary Table 5.
Themes and example quotations for single interventions to prevent falls

Theme	Example quotations			
**Patient education**	"A **tailored education** on falls prevention should be delivered to all hospitalised older adults (≥65 years of age) and other high-risk groups... The individual's cognitive status (i.e. delirium or dementia) should be considered when implementing the education programmes. Use of several education modes (e.g. face-to-face discussions, handouts, videotapes) should be considered."[67] (Montero-Odasso 2022)	"Patients at risk of falling **should be informed** about fall risks and **receive training and advice**…"[62] (Schoberer 2022)<br><br>"A significant effect of patient **education and counselling** regarding falls... a strong recommendation for patients without cognitive impairment and a weak recommendation for residents with cognitive impairment based on the observed effect and the level of confidence in the evidence."[62] (Schoberer 2022)	"Hospital staff should **educate** older adults and provide information about footwear features that may reduce the risk of falls."[66] (Winnipeg 2011)	"The most promising multifactorial interventions in hospital settings tend to involve the education of staff, individualized assessment and education of patients, along with ongoing targeted communication between staff and patients."[70] (WHO 2021)
**Staff education**	"The grading panel argued that the importance of this intervention permitted the assignment of a weak recommendation for **staff education** on fall prevention measures."[62] (Schoberer 2022)	"Health-care organizations provide ongoing **organization-wide education to all staff** in conjunction with other activities to help prevent falls and reduce injuries among persons in their care."[63] (RNAO 2017)	"**All staff are educated on falls prevention** …. Education is ongoing and includes brief understanding of the assessment tool and the implications and strategies for falls prevention. All staff should be aware of environmental indicators that can be a potential hazard to patient safety/ falls."[71] (Degelau 2012)	"**Education for Staff** – All health care providers working with older adults shall be knowledgeable and competent in falls risk assessment, intervention, and prevention."[66] (Winnipeg 2011)

**Delirium and dementia**	"**Delirium, cognitive impairment and dementia** are independent risk factors for falls in older adults in hospital settings…When delirium, dementia and cognitive impairment are managed well, falls are less prevalent [109]. Adapting the environment to promote safety and educating caregivers in strategies for safe mobility can also be of benefit in older adults with delirium. There is some evidence that staff education can help to reduce falls of hospitalised older adults experiencing delirium [110, 111]."[67] (Montero-Odasso, 2022)	Ensure that any multifactorial assessment identifies the patient's individual risk factors for falling in hospital that can be treated, improved or managed during their expected stay. **These may include cognitive impairment.**"[64, 65] (NICE 2013)	"All patients over the age of 65 years on admission, regardless of admitting diagnosis, **should be assessed for both delirium and dementia**… Recognition of delirium is particularly important as a modifiable risk factor for falls."[71] (Degelau 2012)<br><br>"Recognition of **delirium is particularly important** as a modifiable risk factor for falls, and an interdisciplinary approach is needed to screen patients."[71] (Degelau 2012)	"Patients presenting to hospital with an acute change in cognitive function should be **assessed for delirium** and the underlying cause of this change."[72] (ACSQHC 2009)
**Footwear**	"…there is currently **no robust research evidence** to recommend the use of… (ii) **grip socks/nonslip socks** for the purpose of falls prevention."[67] (Montero-Odasso 2022)	"Older people in hospital **should wear sturdy, enclosed shoes** while walking around, rather than socks or slide-on footwear which may be slippery or pose a trip hazard."[70] (WHO 2021)	"… **screen older adults for ill-fitting or inappropriate footwear** (e.g. slippers with no heel counter), foot pain or other foot problems upon admission to hospital. "Encourage older adults to wear proper fitting footwear when mobilizing in hospital."[66] (Winnipeg 2011)	
**Medication review**	"Assess for fall history and the risk of falls **before prescribing potential fall risk increasing drugs (FRIDs)** to older adults…"[67] (Montero-Odasso 2022)	"Older people in hospital should **wear sturdy, enclosed shoes** while walking around, rather than socks or slide-on footwear which may be slippery or pose a trip hazard."[70] (WHO 2021)	"… screen older adults for **ill-fitting or inappropriate footwear** (e.g. slippers with no heel counter), foot pain or other foot problems upon admission to hospital. "Encourage older adults to wear proper fitting footwear	

			when mobilizing in hospital."[66] (Winnipeg 2011)	
**Alarms and sensor mats**	"**The overall rating of the evidence is very low** for both settings. However, due to the practicability of the intervention and possibly more rapid treatment for patients who have fallen, the grading panel decided to issue a weak recommendation for alarm and sensor devices."[62] (Schoberer 2022)	"Monitoring alarms and devices **do not replace the need for safety checks** or regular monitoring by staff."[66] (Winnipeg 2011)	"The bed alarm device aids in alerting the healthcare workers on a high-risk faller's unsafe activity, e.g. getting out of bed without assistance… However, the **results were not statistically significant** for the workgroup to make recommendations regarding its use in the guideline."[69] (Singapore Ministry of health 2006)	
**Continence**	"Enquire about **urinary symptoms** as part of a multifactorial falls risk assessment."[67] (Montero-Odasso 2022)	"Managing **problems with urinary tract function** is effective as part of a multifactorial approach to care… As part of multifactorial intervention, toileting protocols and practices should be in place for older adults at risk of falling… incontinence should be screened in hospital as part of a falls risk assessment."[66] (Winnipeg 2011)	"Interventions to minimise risk for falling associated with **altered urinary**…"[69] (Singapore Ministry of Health 2006)	
**Nutrition**	"**Vitamin D supplementation** to prevent falls should be reserved for those at risk of vitamin D deficiency… nutritional assessment including vitamin D."[67] (Montero-Odasso 2022)	"**Vitamin D and calcium supplementation are** recommended as an intervention strategy to prevent falls in older adults. Benefits from supplementation are most likely to be seen in older adults who have **vitamin D insufficiency or deficiency**, comply with the medication,	"To screen for possible **vitamin D deficiency**, dieticians, nutritionists or health professionals can collect information on the patient's eating habits, food preferences, meal patterns, food intake and sunlight exposure. Alternatively, a blood	

		and respond biochemically to supplementation."[66] (Winnipeg 2011)	sample can be taken."[72] (ACSQHC 2009)	
**Dizziness, vertigo, and syncope**	"Older adults who report '**dizziness**' may have pre-syncope, postural disequilibrium, or gait or balance disorders…"[66] (Winnipeg 2011)	"Patients who report **unexplained falls or episodes of collapse** should be assessed for the underlying cause… Patients with unexplained falls or episodes of collapse who are diagnosed with the cardioinhibitory form of carotid sinus hypersensitivity should be treated by inserting a dual-chamber cardiac pacemaker."[72] (ACSQHC 2009)		
**Environment**	"To ensure a **safe environment**: implement universal falls precautions; identify and modify equipment and other factors in the physical/structural environment that contribute to risk for falls and fall injuries…"[63] (RNAO 2017)	"Ensure that all aspects of the **patient's environment** (including flooring, lighting, furniture and accessories such as handholds) that may affect the patient's risk of falls are identified and resolved."[68] (Gea 2015)	"… **cleaning up** the area around the bed, managing the hospital environment, checking pagers, and identifying other patient needs."[73] (Cho 2020)	"**Physical hazards** are often involved in patient falls. An environmental assessment or checklist can often identify modifiable risk factors to falls, such as floor mats, lack of handrails in bathrooms, poorly anchored rugs or clutter."[71] (Degelau 2012)<br><br>"… confirm that hallways and patient areas are well lit, uncluttered and free of spills – also that locked doors are kept locked when unattended, handrails are secure, and tables and chairs are sturdy."[71] (Degelau 2012)

Supplementary Table 6.
Themes and example quotations for prevention and management (multifactorial interventions)

Theme	Example quotations				
**Multifactorial interventions**	"Personalised single or **multidomain falls prevention strategies** based on identified risk factors, behaviours or situations should be implemented for all hospitalised older adults (≥65 years of age), or younger individuals identified by health professionals as at risk of falls."[67] (Montero-Odasso 2022)	"**Multifactorial interventions** based on individual risk factors significantly reduce falls and are strongly recommended in the hospital."[62] (Schoberer 2022)	""Ensure that any **multifactorial assessment identifies individual risk factors** that can be treated or improved during the expected patient stay. These may include: Cognitive impairment; Incontinence problems; Inappropriate footwear or lack of footwear; History of falls, including causes and consequences ...; Medication; Health problems that may increase the risk of falls; Postural instability, mobility problems or balance problems; Visual deficit; Vasovagal syndrome."[68] (Gea 2015)	"**Multi-factorial interventions** (which may include exercise) appear to be effective at reducing hospital falls."[74] (AGILE 2012)	"**Multifactorial interventions** that increase observation and surveillance have been found to be effective on falls."[71] (Degelau 2012)

Supplementary Table 7.
Themes and example quotations for documentation of falls and associated injuries

Theme	Example quotations		
**Systems for documentation of falls and associated injuries**	"Process **quality indicators** include the assessment and documentation of fall risk factors and multidisciplinary case discussions in hospital settings. Outcome quality indicators belong, for example, to a lowered fall rate and reduced injuries caused by falls."[62] (Schoberer 2022)	"**Collect data** on falls among older people in hospital: **Monitor administrative and clinical data** (e.g. falls per 1000 occupied bed days, fall rates by type of falls, specific location of falls) and investigate the frequency and severity of falls (e.g. injury rate, injury rate by severity) in the health organization."[70] (WHO, 2021)	"**Organizational leadership** needs to support systems that **promote learning, ongoing evaluation and improvement** of the falls prevention program including analysis of falls rates and injuries (falls/1,000 patient days and falls with injury/1,000 patient days). The analysis should report on the internal effectiveness (validity) of falls screening and effectiveness of interventions applied to those screened at risk."[71] (Degelau, 2012)

Supplementary Table 8.
Themes and examples of quotations on barriers to implementation

Theme	Example quotations		
**Barriers to implementation**	"In considering **implementation of** the Guidelines at a local level, healthcare professionals are encouraged to **identify the barriers, enablers and facilitators to evidence-based practice** within their own environment and determine the best strategy for local needs. Where change is required, initial and ongoing education is essential and is relevant to all recommendations in the Guidelines."[75] (Stroke Foundation, 2024)	"**Successful implementation** requires a **supportive organisational context**, which should be assessed prior to implementing an organisational change addressing falls. Tools exist to help guideline implementers assess context."[67] (Montero-Odasso 2022)	Quote: "Based on the multifaceted and tailored implementation strategies used, **eight recommendations for the guideline implementation** can be made…"[62] (Schoberer 2022)

References for Supplementary Files

1. Lung Foundation Australia. COPD-X Concise Guide. Milton, Queensland, Australia. 2024. Available from: https://lungfoundation.com.au/resources/copd-x-concise-guide/.
2. Agency for Healthcare Research and Quality. Preventing falls in hospital toolkit. 2024; Available from: https://www.ahrq.gov/patient-safety/settings/hospital/fall-prevention/toolkit/index.html.
3. EuroSafe. Available from: https://www.eurosafe.eu.com/publication/policy-briefing.
4. Australian Commission on Safety and Quality in Healthcare. The National Safety and Quality Health Service Standards. 2024. Available from: https://www.safetyandquality.gov.au/standards/nsqhs-standards.
5. Camicioli, R., Morris, M. E., Pieruccini-Faria, F., Montero-Odasso, M., Son, S., Buzaglo, D., . . . Nieuwboer, A. Prevention of Falls in Parkinson's Disease: Guidelines and Gaps. Movement Disorders Clinical Practice. 2023;10(10) 1459-1469.
6. Callus C, Bonnici M, Grech M, Vassallo MA. The development of an in-patient fall acute management guideline. European Geriatric Medicine. 2022;13 (Supplement):S236-S7.
7. Ellen F, Toby E, Chris T, Lisa M, David H, Rixt ZGA, et al. Global guidelines for falls in older adults: Working group 12: Fear of falling. European Geriatric Medicine. 2022;13 (Supplement):S239.
8. Todd RH, Susan; Bell, Sue. Clinical practive guideline for preventing falls in geriatric patients. University MW. Minnesota2022.
9. Carroll Smith W. Fall Clinical Practical Guideline in a Psychiatric Hospital. University W. Walden Online University 2022.
10. Association AD Professional Practice Committee: Standards of Medical Care in Diabetes. Diabetes Care. 2021;45 (Supplement):S3-S.
11. EBPracticenet Working Group. Fall prevention in elderly persons with an increased risk of falling (Occupational Therapy Guideline). Belgium 2021.
12. Fraenkel L, Bathon JM, England BR, St.Clair EW, Arayssi T, Carandang K, et al. 2021 American College of Rheumatology Guideline for the Treatment of Rheumatoid Arthritis. Arthritis Care & Research. 2021;73(7):924-39.
13. Cho I, Kim J, Chae JS, Jung M, Kim YH. Development of ICNP-based inpatient falls prevention catalogue. International Nursing Review. 2020;67(2):239-48.
14. College of Occupational Therapy. Occupational therapy in the prevention and management of falls. Practice Guideline 2020.
15. Johnson K, Scholar H, Stinson K, Nea B, Sherry Razo MAL. Patient fall risk and prevention strategies among acute care hospitals. Applied Nursing Research. 2020;51:151188.
16. Lamb SE, Bruce J, Hossain A, Ji C, Longo R, Lall R, et al. Screening and Intervention to Prevent Falls and Fractures in Older People. N Engl J Med. 2020;383(19):1848-59.
17. Ocker SA, Barton SA, Bollinger N, Leaver CA, Harne-Britner S, Heuston MM. Preventing Falls Among Behavioral Health Patients. Am J Nurs. 2020;120(7):61-8.

18. Araki E, Goto A, Kondo T, Noda M, Noto H, Origasa H, et al. Japanese Clinical Practice Guideline for Diabetes 2019. Diabetology International. 2020;11(3):165-223.
19. Kolasinski SL, Neogi T, Hochberg MC, Oatis C, Guyatt G, et al. 2019 American College of Rheumatology/Arthritis Foundation Guideline for the Management of Osteoarthritis of the Hand, Hip, and Knee. Arthritis & Rheumatology. 2020;72(2):220-33.
20. Kim C, Sung J, Lee JH, Kim WS, et al. Clinical Practice Guideline for Cardiac Rehabilitation in Korea: Recommendations for Cardiac Rehabilitation and Secondary Prevention after Acute Coronary Syndrome. Korean Circ J. 2019;49(11):1066-111.
21. Health TNBo. National clinical guideline for the prevention of falls in the elderly. Authority TDH. Copenhagen. 2018.
22. Malcolm J, Halperin I, Miller DB, Moore S, Nerenberg KA, Woo V, et al. In-Hospital Management of Diabetes. Canadian Journal of Diabetes. 2018; 42:S115-S23.
23. Kenny RA, Romero-Ortuno R, Kumar P. Falls in older adults. Medicine. 2017;45(1):28-33.
24. Kim K-I, Jung H-K, Kim CO, Kim S-K, Cho H-H, Kim DY, et al. Evidence-based guidelines for fall prevention in Korea. The Korean Journal of Internal Medicine. 2017;32(1):199.
25. Lee R. The CDC's STEADI initiative: Promoting older adult health and independence through fall prevention. American Family Physician. 2017;96(4):220-1.
26. Morris R, O'Riordan S. Prevention of falls in hospital. Clinical Medicine. 2017;17(4):360-2.
27. Pearce L. Preventing falls in hospital. Nursing Management. 2017;23(10):11.
28. American Geriatrics Society. AGS/BGS Clinical Practice Guideline: Prevention of falls in older persons. Summary of recommendations. 2016.
29. Williams R. Guide to ensure older people avoid trips and falls in hospital. Nursing Management. 2016;23(8):11.
30. Crandall M, Duncan T, Mallat A, Greene W, Violano P, Christmas AB, et al. Prevention of fall-related injuries in the elderly: an Eastern Association for the Surgery of Trauma practice management guideline. Journal of Trauma and Acute Care Surgery 2016;81(1):196-206. 2016.
31. Lee JH, Kim HA, Park SW. Prevention of fall in the hospital. Journal of the Korean Medical Association. 2015;58(2):123-30.
32. Falls in older people: assessing risk and prevention. London: National Institute for Health and Care Excellence; 2015.
33. Barker W. Assessment and prevention of falls in older people. Nursing Older People. 2014;26(6):18-24.
34. Keus SHJ MM, Graziano M, el. European Physiotherapy Guideline for Parkinson's disease. The Netherlands: KNGF/ParkinsonNet; 2014.
35. Swift CG, Iliffe S. Assessment and prevention of falls in older people: concise guidance. Clinical Medicine. 2014;14(6):658-62.
36. Tung EE, Newman JS. Fall prevention in hospitalized patients. Hospital Medicine Clinics. 2014;3(2):e189-e201.
37. van der Marck MA, Klok MPC, Okun MS, Giladi N, Munneke M, Bloem BR, et al. Consensus-based clinical practice recommendations for the examination and management of falls in patients with Parkinson's disease. Parkinsonism & Related Disorders. 2014;20(4):360-9.

38. Stevens JA. The STEADI Tool Kit: A Fall Prevention Resource for Health Care Providers. IHS Prim Care Provid. 2013;39(9):162-6.
39. Stevens JA, Phelan EA. Development of STEADI: A fall prevention resource for health care providers. Health Promotion Practice. 2013;14(5):706-14.
40. Chow WB, Rosenthal RA, Merkow RP, Ko CY, Esnaola NF. Optimal preoperative assessment of the geriatric surgical patient: A best practices guideline from the American College of Surgeons national surgical quality improvement program and the American Geriatrics Society. Journal of the American College of Surgeons. 2012;215(4):453-66.
41. Health Care Association of New Jersey. Fall management guideline. 2012.
42. Ummenhofer W, Suhm N. Fractured neck of femur: Guidelines and beyond. Anaesthesia. 2012;67(1):2-4.
43. Vance J. The clinical practice guideline for falls and fall risk. Transl Behav Med. 2012;2(2):241-3.
44. American Geriatrics Society, British Geriatrics Society, Kenny RAM, Rubenstein LZ, Tinetti ME, Brewer K, et al. Summary of the Updated American Geriatrics Society/British Geriatrics Society Clinical Practice Guideline for Prevention of Falls in Older Persons. Journal of the American Geriatrics Society. 2011;59(1):148-57.
45. Beauchet O, Dubost V, Revel-Delhom C, Berrut G, Belmin J. How to manage recurrent falls in clinical practice: guidelines of the French Society of Geriatrics and Gerontology. The Journal of Nutrition, Health & Aging. 2011;15:79-84.
46. Bradley SM. Falls in older adults. Mount Sinai Journal of Medicine. 2011;78(4):590-5.
47. Kline NE, Davis ME, Thom B. Fall risk assessment and prevention. Oncology (Williston Park, NY). 2011;25:17-22.
48. American Geriatrics Society. AGS/BGS clinical practice guideline: prevention of falls in older persons. 2010.
49. Handoll H. Prevention of falls and fall related injuries in older people in nursing homes and hospitals. Injury prevention. Journal of the International Society for Child and Adolescent Injury Prevention. 2010;16(2):137-8.
50. Oliver D. Preventing falls and falls-injuries in hospitals and long-term care facilities. Reviews in Clinical Gerontology. 2007;17(2):75-91.
51. Naqvi F, Lee S, Fields SD. An evidence-based review of the NICHE guideline for preventing falls in older adults in an acute care setting. Geriatrics. 2009;64(3).
52. Loew F, Maupetit C. Preventing falls and fractures today. Revue Medicale Suisse. 2005;1(11):781-4.
53. Association American Medical Directors. Falls and fall risk clinical practice guideline. Columbia, MD. 2003.
54. Moreland J, Richardson J, Chan DH, O'Neill J, Bellissimo A, Grum RM, et al. Evidence-based guidelines for the secondary prevention of falls in older adults. Gerontology. 2003;49(2):93-116. 2003.
55. Carson M, Cook J. A strategic approach to falls prevention. British Journal of Clinical Governance. 2000;5(3):136-41.
56. Feder G, Cryer C, Donovan S, Carter Y. Guidelines for the prevention of falls in people over 65. The Guidelines' Development Group. BMJ. 2000;321(7267):1007-11.
57. Rutledge DN, Donaldson NE, Pravikoff DS. Fall risk assessment and prevention in healthcare facilities. Online Journal of Clinical Innovations. 1998;1(9):1-33.

58. Baraff LJ, Della Penna R, Williams N, Sanders A. Practice Guideline for the ED Management of Falls in Community-Dwelling Elderly Persons. Annals of Emergency Medicine. 1997;30(4):480-92.
59. Clinical Care Standards. Australian Commission on Safety and Quality in Health Care; 2022; Available from: https://www.safetyandquality.gov.au/sites/default/files/2022-08/clinical_care_standards_-_faqs_for_clinicians.pdf.
60. Buse K, Mays N, Walt G. Making Health Policy. United Kingdom: McGraw-Hill Education; 2012.
61. Thoele K, Ferren M, Moffat L, Keen A, Newhouse R. Development and use of a toolkit to facilitate implementation of an evidence-based intervention: a descriptive case study. Implementation Science Communications. 2020;1(1):86.
62. Schoberer D, Breimaier HE, Zuschnegg J, Findling T, Schaffer S, Archan T. Fall prevention in hospitals and nursing homes: Clinical practice guideline. Worldviews on evidence-based nursing. 2022;19(2):86-93.
63. Registered Nurses' Association of Ontario. Preventing Falls and Reducing Injury from Falls. Toronto, ON. 2017.
64. NICE. Falls in older people: assessing risk and prevention: National Institute for Health and Care Excellence. 2013.
65. 2019 surveillance of falls in older people: assessing risk and prevention: NICE National Institute for Health Care Excellence. 2019.
66. Fall prevention and management. Regional Clinical Practice Guidelines: Acute care facilities, Personal care homes, long term care facilities, community services & programs: Winnipeg Regional Health Authority. 2011.
67. Montero-Odasso M, van der Velde N, Martin FC, Petrovic M, Tan MP, Ryg J, et al. World guidelines for falls prevention and management for older adults: a global initiative. Age Ageing. 2022;51(9).
68. Gea LB, Telleria BG, Martinez JA, Arnaiz EM, Gonzalez LP. Evidence-based recommendations for Prevention and Treatment of falls. Osteba. Departmento de Salud (Spain). 2015. Available from: https://regroup-production.s3.amazonaws.com/documents/ReviewReference/764967090/4
69. Ministry of Health Nursing Clinical Practice Guidelines 1/2005. Prevention of falls in hospitals and long term care institutions. Singapore Nursing Journal. 2006;33(2):52-4.
70. Step Safely: Strategies for preventing and managing falls across the life-course. Geneva: World Health Organization; 2021. Licence: CC BY-NC-SA 3.0 IGO.
71. Degelau J, Belz M, Bungum L, Flavin PL, Harper C et al. Prevention of falls (acute care). Institute for Clinical Systems Improvement Health Care Protocol Update 2012.
72. Preventing falls and harm from falls in older people: best practice guidelines for Australian hospitals: Australian Commission on Safety Quality in Healthcare. 2009.
73. Cho I, Park KH, Suh M, Kim EM. Evidence-based Clinical Nursing Practice Guideline for Management of Inpatient Falls: Adopting the Guideline Adaptation Process. J Korean Acad Fundam Nurs. 2020; 27(1):40-51.
74. AGILE. Guidelines for the physical therapy management of older people at risk of falling: Chartered Physiotherapists Working with Older People. 2012.
75. Stroke Foundation. Clinical Guidelines for Stroke Management. Available from: https://informme.org.au/guidelines/living-clinical-guidelines-for-stroke-management.
